# Navigating idiopathic Chylothorax: a case report and review of the diagnosis and management

**DOI:** 10.1093/omcr/omaf173

**Published:** 2025-10-22

**Authors:** Kyle G Alexander, Elena Tsavala, Ivy Mensah, Polyxeni Ntontsi, Ioannis Hatzimanolis, Evgenios Metaxas

**Affiliations:** Department of Basic and Clinical Sciences, University of Nicosia Medical School, 46 Makedonitissas Avenue, CY-2417, Nicosia, Cyprus; Department of Basic and Clinical Sciences, University of Nicosia Medical School, 46 Makedonitissas Avenue, CY-2417, Nicosia, Cyprus; Department of Basic and Clinical Sciences, University of Nicosia Medical School, 46 Makedonitissas Avenue, CY-2417, Nicosia, Cyprus; Department of Pulmonology, Limassol General Hospital, Limassol, Nikaias, Polemidia Kato, Limassol, Cyprus; Department of Pulmonology, Limassol General Hospital, Limassol, Nikaias, Polemidia Kato, Limassol, Cyprus; Department of Basic and Clinical Sciences, University of Nicosia Medical School, 46 Makedonitissas Avenue, CY-2417, Nicosia, Cyprus

**Keywords:** Chylothorax, decortication, idiopathic, lymphangiography, thoracic duct ligation

## Abstract

We present a case of a 31-year-old male patient with Idiopathic chylothorax and concomitant pneumothorax. He presented with new-onset chest discomfort, shortness of breath, and dry cough. After establishing the diagnosis and following attempted conservative management, the patient experienced chylous drainage exceeding 1 L/day and substantial weight loss. Extensive pleural fluid and serum analysis failed to identify the underlying cause, and lymphangiography revealed no point of leakage. The patient ultimately required invasive duct ligation through thoracotomy and subsequent decortication for resolution of symptoms. This case underscores the challenges of diagnosing and managing idiopathic chylothorax and highlights the need for comprehensive approaches to such cases.

## Background

Chylothorax is a medical condition, defined as the accumulation of chyle within the pleural cavity [1]. Chyle, a milky fluid containing lymph and chylomicrons, is transported through the lymphatic system via various tributaries, notably the thoracic duct which is situated in the posterior mediastinum. Chylothorax occurs when there is a disruption or obstruction in such vessels, leading to the leakage of chyle into the pleural space [1].

Aetiologies of chylothorax include: Trauma, frequently of iatrogenic origin, such as cardiothoracic surgeries. Malignancies, including lymphomas, which cause chylothorax either through infiltration or compression of the lymphatic system. Congenital malformations of the lymphatic system, infections such as tuberculosis, and multisystem disorders including sarcoidosis or rheumatoid arthritis are also documented as causes of chylothorax [2–4]. In some cases, no clear etiology is identified—known as idiopathic chylothorax [1].

Patients with chylothorax present with symptoms similar to pleural effusion, including dyspnea, cough, and chest discomfort. As a result of chyle’s composition rich in lipids and lymphocytes, individuals may also exhibit weight loss and frequent infections due to nutritional and immuno-deficiency respectively. Physical examination classically reveals decreased breath sounds, dullness to percussion, and decreased tactile vocal fremitus on the side of the chylothorax [5].

The diagnosis of chylothorax involves the combination of clinical examination, blood work, radiological imaging, and biochemical analysis of pleural fluid. Chest X-rays, ultrasound, or computed tomography (CT) scans confirm pleural effusion and on occasion provide information regarding underlying cause [6]. Pleurocentesis and analysis of the pleural fluid is required for diagnosis, typically revealing a milky appearance, high triglyceride levels (>110 mg/dL), and chylomicrons on microscopy [7]. Additional investigations, including thoracic duct lymphangiography and magnetic resonance imaging (MRI), can be conducted to identify the anatomical site of chyle leakage/obstruction [6].

Chylothorax management involves three aims: to relieve symptoms, correct deficiencies, and promote the cessation of chyle leakage. Initial conservative management includes dietary modifications and the use of medium-chain triglyceride (MCT) supplements to bypass the lymphatic system [8], and somatostatin analogues such as octreotide (extra). In the event conservative management fails to resolve symptoms or in cases of large chylothoraces, more invasive interventions include surgical repair, thoracic duct ligation or embolization [9]. In some instances, pleurodesis can be performed to prevent chyle accumulation [10]. Close monitoring and follow-up are crucial to evaluate treatment response and manage potential complications.

While the diagnostic and management principles for chylothorax are well established, there is limited literature on idiopathic cases. This case report aims to highlight such idiopathic chylothorax, assisting physicians to follow a structured diagnostic approach and management strategies in the absence of clear etiology.

## Case presentation

A 31-year-old male patient presented to the emergency department with new-onset chest discomfort and shortness of breath, accompanied by an insidious onset dry cough over the past week. No signs of respiratory failure were present, with an SpO2 reading of 96%. He denied any direct chest trauma, surgical history, immobilization, recent illness or travel history. Past medical history was significant for pneumonia and herpes zoster infection, fifteen and four years ago respectively. No allergies or weight loss were reported. A thorough family history was obtained, revealing no known hereditary or congenital conditions, such as lymphatic malformations or genetic syndromes. Physical examination revealed low-grade fever, reduced tactile vocal fremitus, and dullness to percussion over the right lung. Auscultation findings included decreased lung sounds and negative egophony in the mid-to-lower regions of the right lung. The patient was normotensive, with unremarkable cardiovascular and abdominal exams.

## Investigations

Initial laboratory studies revealed unremarkable full blood count, renal and liver function tests and lipoprotein profile. Arterial blood gas samples were consistent with a mild respiratory alkalosis.

A chest x-ray indicated a right-sided pleural effusion, alongside fluid buildup in the right minor fissure and atelectatic changes in the right lower lung field.

**Figure 1:**
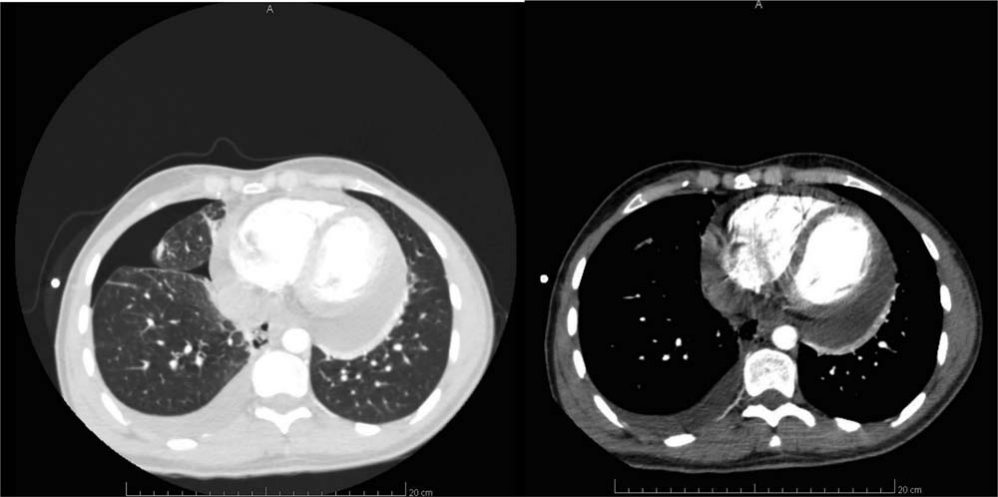
A and B. CT scan of the thorax: Right-sided pleural effusion with chest drain, pneumothorax and pneumomediastinum (left), pericardial fluid collection (right).

CT scan of the thorax confirmed a right-sided pleural effusion, with concomitant pneumothorax and pneumomediastinum (Fig. 1A). No significant parenchymal changes were observed in the lungs, including signs of consolidation, ground-glass opacities, or atelectasis. A pericardial effusion at the base of the heart was also visualized (Fig. 1B).

CT pulmonary angiogram (CTPA) ruled out the presence of pulmonary emboli. Abdominal and pelvic CT revealed mild fatty liver infiltration, but was otherwise insignificant. A subsequent echocardiogram confirmed the existence of pericardial effusion, without evidence of tamponade.

**Figure 2:**
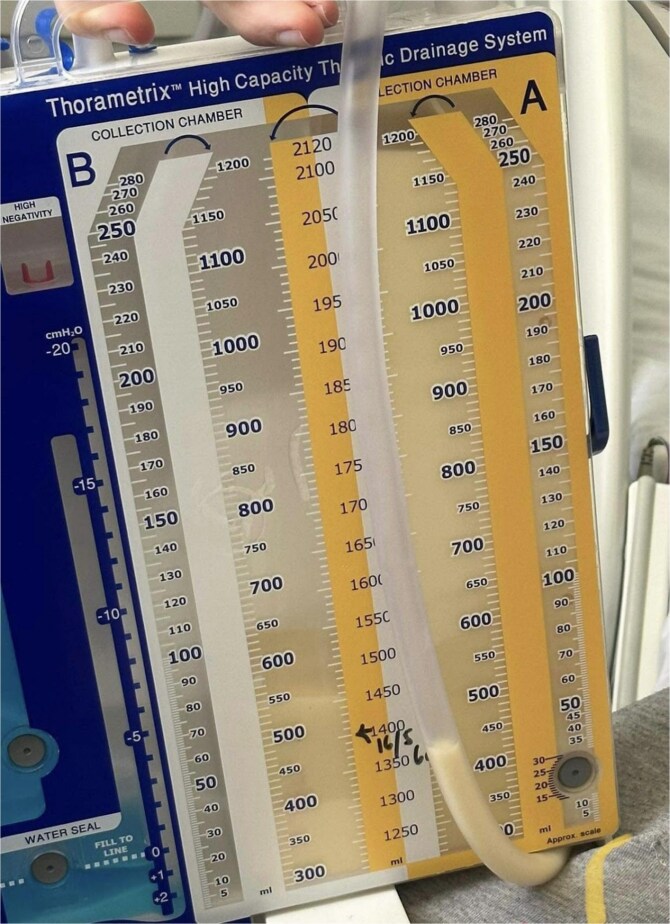
Pleural fluid drainage, exhibiting a milky appearance.

The total volume of drained pleural fluid was 14 250 mL. The pleural fluid drained was milky yellow in appearance (Figure 2). Over the duration of hospital admission. Pleural fluid analysis (Table 1) showed a total cholesterol level below 3 mg/dl and a triglyceride level of 190 mg/dl, confirming the diagnosis of chylothorax. Microbiological examination of the pleural fluid was performed; acid-fast bacilli were not observed on Ziehl-Neelsen staining, and mycobacterial cultures were negative. Cytological examination revealed chronic non-specific inflammation, and flow cytometry for CD3, CD4:CD8, CD19, CD20 was unsuspicious for lymphoma.

**Table 1:** Lab values of pleural fluid analysis.

Pleural Fluid Content	Result
White blood cells	850/ul
Erythrocytes	900/HI
Neutrophils	18%
Lymphocytes	80%
Total cholesterol	95 mg/dl
LDL cholesterol	3 mg/dl
Triglycerides	190 mg/dl
Glucose	95 mg/dl
Lactate dehydrogenase	212 U/l
Albumin	3.8 g/dl
Biuret	5.0 g/dl

A comprehensive immunological panel was unremarkable, including antinuclear antibody (ANA), Rheumatoid Factor (RF), anti 32GPI, anti PR3, anti MPO, anti dsDNA, anti Smith (Sm), anti Jo-1, anti centromere, anti CCP, anti Cardiolipin, anti SSA/SSB and levels of immunoglobulin (Ig), Angiotensin converting enzyme (ACE) and C3/C4. Cancer markers were negative, including Alpha fetoprotein (AFP), Alkaline Phosphatase (ALP), Carcinoembryonic Antigen (CEA), CA 19.9, CA 125, CA 15.3, CYFRA21-1, Calcitonin, Prostate specific antigen (PSA), Beta Microglobulin. Hepatitis B and C Viruses (HBV and HCV) and Human Immunodeficiency Virus (HIV) antibodies were non-reactive. Urine free light chains were negative. IgG4 levels were within reference range.

## Treatment

On the first day of hospitalization empirical antibiotics were administered for the working diagnosis of pneumonia with parapneumonic effusion, including intravenous (IV) tavanic acid 500 mg and ceftriaxone 2 g. The patient’s dyspnea and chest discomfort improved significantly with drainage of the effusion. Once the diagnosis of Chylothorax was established, total parenteral nutrition (TPN) was started for eight days alongside IV normal saline (0.9%). On the 8th day of admission the patient started a zero fat diet and was administered IV octreotide 150 μg increasing to 200 μg over 5 days. Subcutaneous enoxaparin was used to prevent venous thromboembolism. Cardiovascular evaluation was conducted for the pericardial effusion, with a non-steroidal anti-inflammatory drug (Brufen) prescribed for eighteen days.

Conservative management was continued for 3 weeks, with persistent chylous drainage averaging 1 L/day and a 12 kg weight loss. Subsequent lymphangiography was unremarkable, with no leakage point identified. The patient was referred to a specialized cardiothoracic surgery unit, where ligation of the thoracic duct was performed via open thoracotomy, alongside right-sided lung decortication. During the procedure, the surgeon assessed the thoracic duct anatomy and chose to ligate a section based on the typical anatomical locations where injuries and obstructions commonly occur, as well as the observed chyle accumulation. Chemical pleurodesis was not performed during the thoracic duct ligation. The patient remained hospitalized for 24 days, with CT scans confirming gradual resolution of the chylothorax. Residual pneumomediastinum was noted following the procedure; however, no evidence of recurrent chyle accumulation was observed. The patient has since been followed up in the Limassol General Hospital outpatient clinic, where follow-up X-rays and blood tests have shown stable findings. There has been no recurrence of symptoms, and the patient’s weight remains stable.

## Discussion

This case report presents a young male patient with shortness of breath, dry cough, and chest discomfort. The report provides an investigatory approach to reach the diagnosis of chylothorax, which in some cases can lack a clear etiology or source of leak. Idiopathic chylothorax, characterized by the accumulation of chyle in the pleural space without an identifiable cause after thorough evaluation, accounts for approximately 6–15% of cases [11]. In children, especially neonates, idiopathic cases are more common and may relate to congenital lymphatic abnormalities or birth trauma, while in adults, ruling out occult trauma or malignancy is most crucial before reaching the diagnosis [12]. Ultimately, we highlight the importance of diagnostic algorithms for pleural effusions, especially in idiopathic cases where limited history and no clear cause can delay diagnosis.

Following clinical examination, imaging and thoracocentesis, the diagnosis of right-sided chylothorax was confirmed in this case. Chest X-ray also revealed a pneumothorax on the same side. In the absence of trauma or previous thoracic surgery, further blood tests and pleural fluid analysis were required to establish etiology. Flow cytometry and cancer markers were essential to rule out malignancies, notably lymphoma. Absence of acid-fast bacilli were not observed on Ziehl-Neelsen staining, ruling out the possibility of pulmonary tuberculosis. Comprehensive immunological testing were carried out to exclude multisystem causes of chylothorax, including sarcoidosis, rheumatoid arthritis and SLE. In the initial absence of lymphangiography, a thoracic duct leak was the presumed etiology.

**Figure 3:**
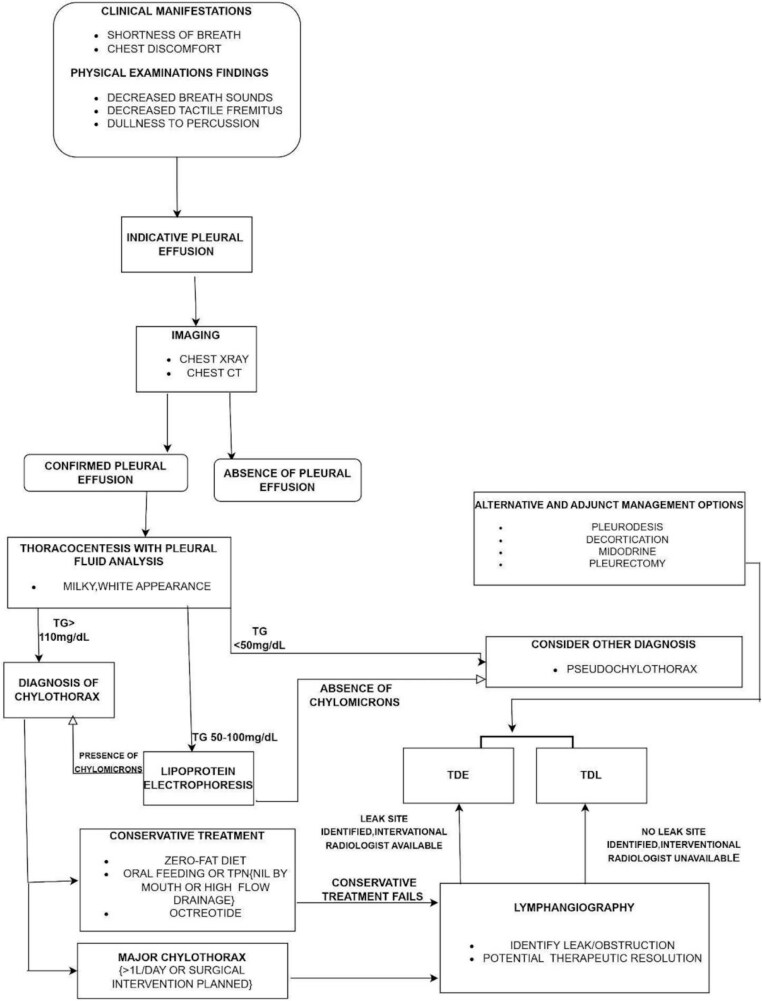
Flow chart highlighting the diagnostic and management approach to Chylothorax.

A summary of the approach to reach the diagnosis of chylothorax is highlighted in Fig. 3, alongside Table 2, which reviews the purpose, benefits and limitations of each investigation.

**Table 2:** Investigations for the diagnosis of Chylothorax.

Investigation	Clinical Purpose	Benefits	Limitations
Chest X-ray	Assess presence of pleural effusion	Readily available/inexpensive	Cannot always distinguish between atelectasis and pleural effusion. Cannot distinguish Chylous effusion
Chest CT	Further categorize pleural effusion	Potential to differentiate chylothorax from other effusions. Can reveal underlying etiology.	Rarely definitively identifies chylothorax.
MRI	Further categorize pleural effusion	Can differentiate chylothorax from other effusions. Can identify underlying etiology. Does not use ionizing radiation.	Costly and time-consuming procedure
Pleural fluid analysis: Visual inspection	Identify chylothorax based on milky-white appearance	Quick and simple	Not sensitive or specific (seen in pseudochylothorax and empyema)
Pleural fluid analysis: TG concentration	To diagnose chylothorax	High diagnostic accuracy when TGs > 110 mg/dl	Non-chylous effusions may rarely present with elevated TGs
Pleural fluid analysis: Lipoprotein electrophoresis	To diagnose chylothorax (reserved for cases with borderline TG concentration)	Most specific and sensitive investigation for diagnosis	Not widely available and expensive
Lymphangiography	To identify sites of leakage prior to surgery. Confirms diagnosis of chylothorax.	Direct visualization of the lymphatic system. Resolution of leakage in some cases.	Invasive procedure with significant complications and contraindications. Not widely available.
Lymphoscintigraphy	To identify sites of leakage prior to surgery. Confirms diagnosis of chylothorax.	Non invasive procedure to visualize the lymphatic system.	Exposure to radiotracer. Not widely available.

The treatment and management strategies employed to address chylothorax can vary depending on the underlying cause, severity of symptoms, and the patient’s overall health status. The primary goal of treatment is to control the chyle leakage, reduce pleural fluid accumulation, and promote the resolution of symptoms. Conservative management strategies were attempted initially, as according to the British Thoracic Society guidelines, which included pleural fluid drainage, TPN, and octreotide, followed by a zero fat diet. There are no formal guidelines as to the duration of time conservative therapy can be tried before invasive procedures are indicated, however 14 days has been suggested as a maximum limit for low-volume leaks and within 7 days for high-volume leaks [13].

Following a three week conservative approach, Chyle continued to drain from the pleural space, warranting investigation with Lymphangiography to identify leaks, obstructions or anatomical abnormalities [14]. As observed in this case, lymphangiography rarely cannot identify the precise site of chyle leakage or obstruction.

There are two procedures utilized to stop leakage of chylous fluid, thoracic duct embolisation (TDE) and surgical ligation (TDL). TDE is a minimally invasive alternative to TDL, with a recent meta analysis finding clinical success rates of 79.4% and complications occurring in 2.4% of patients [15]. TDL through thoracotomy is employed when concurrent thoracic procedures are required, in this case to perform decortication [15]. When chylothorax becomes chronic or recurrent, it can lead to the formation of a fibrous peel (‘pleural peel’), ultimately restricting lung expansion and contributing to persistent leakage of chyle. Decortication is a surgical procedure utilized to remove fibrous peel, and is generally indicated when chylothorax is long-standing or appears loculated on imaging [16].

Ultimately the choice between conservative and surgical approaches depends on factors such as the underlying cause, the patient’s overall health, the severity of symptoms, and the response to initial therapies. A multidisciplinary approach involving pulmonologists, thoracic surgeons, and nutritionists is crucial to determine the most appropriate treatment strategy for each individual case of chylothorax.

The coexistence of chylothorax and pneumothorax on the same side, is a rare phenomenon with limited documented cases in the medical literature, most of which are related to multisystem disorders such as sarcoidosis and leiomyomatosis [17–19]. While the etiology of chylothorax is typically related to thoracic duct injury or obstruction, pneumothorax, which arises from a breach in the visceral or parietal pleura, commonly occurs following trauma or underlying lung diseases such as COPD and asthma. The concurrent occurrence of these pathologies may suggest underlying anatomical abnormalities or predisposing factors that can lead to both conditions.

The presence of both pericardial effusion and chylothorax is well documented and can be explained by the shared mediastinal lymphatic pathways and proximity of the two structures [20]. Both may result from injury to the thoracic duct or lymphatic channels, following trauma, surgery, malignancy, or congenital anomalies [20]. Case reports have reported chyle tracking from one compartment to the other, such as initial chylothorax followed by chylopericardium [21, 22]. However, overall frequency remains poorly defined due to the rarity of these conditions and reliance on case reports such as this.

This case presents a rare and challenging instance of chylothorax that proved to be idiopathic in nature. Despite thorough investigation, including lymphangiography, no identifiable cause or leakage site could be located. This finding underscores the elusive nature of certain chylothorax cases, which can pose significant diagnostic and management challenges for clinicians. Future studies should focus on comparing the diagnostic accuracy and cost effectiveness of different investigations for chylothorax, alongside treatment strategies tailored to the underlying etiology. Such studies would provide more data to guide evidence-based clinical decision-making.

## Conclusion

This case of idiopathic chylothorax in a previously healthy young male underscores the complexity and diagnostic challenges associated with this condition. Despite extensive investigations yielding no identifiable etiology, the multidisciplinary approach adopted in this case facilitated effective management and resolution of symptoms. This report highlights the necessity for clinicians to remain vigilant in their diagnostic processes and consider idiopathic chylothorax as a potential diagnosis when faced with unexplained pleural effusions. Future research should focus on refining diagnostic algorithms and treatment strategies for idiopathic cases to enhance patient outcomes.
